# Coulomb Effect of Intermediate Products of Core–Shell SiO_2_@Al Nanothermite

**DOI:** 10.3390/molecules30040932

**Published:** 2025-02-17

**Authors:** Jinping Zhang, Yuanhong Chu, Fei Wang, Shan Yuan, Minghui Tan, Hui Fu, Yu Jia

**Affiliations:** 1 Henan Provincial Key-Laboratory of Nano-Composite and Applications, Institute of Nanostructured Functional Materials, Faculty of Engineering, Huanghe Science and Technology College, Zhengzhou 450006, China; chuyuanh@163.com (Y.C.); wangf157@hhstu.edu.cn (F.W.); 18737962065@163.com (S.Y.); 17634517502@163.com (M.T.); iefh@hhstu.edu.cn (H.F.); 2 School of Materials Science and Engineering, Henan University of Science and Technology, Luoyang 471023, China; 3 Key Laboratory for Special Functional Materials of Ministry of Education, School of Physics and Electronics, Henan University, Kaifeng 475001, China; 4 Institute of Quantum Materials and Physics, Henan Academy of Science, Zhengzhou 450046, China

**Keywords:** molecular dynamics, reactive force field, reaction process, intermediate products, Coulomb effect

## Abstract

Nanothermites as high-energy-density and high-reaction-rate materials have important applications in civil and military fields. Nevertheless, it is difficult to detect all intermediates and products using conventional experimental methods. In this work, the reaction process of core-shell SiO_2_@Al nanoparticles under adiabatic conditions was investigated through molecular dynamics simulations using a reactive force field (ReaxFF). In the microcanonical ensemble, the redox reaction of SiO_2_@Al nanothermite becomes explosive due to the huge energy release during Al-O bond formation. The gaseous products are mainly the intermediate products Al_5_O and Al_4_O as well as the final products Al_2_O, AlO, Si and Al. Analyses of the steric charge distributions and evolution show that the Coulomb effect causes the number of intermediates Al_5_O (0.32|e|) to increase to the maximum, then slowly decrease and remain stable. But the tetrahedral Al_4_O cluster is almost charge-neutral, at −0.05|e|, and the number remained almost constant. This work is expected to provide deeper insights into the complex reaction mechanism of nanothermite.

## 1. Introduction

Nanothermites, which consist of nanoscale metals and metal oxides (fuel and oxidizer, respectively), have attracted considerable attention due to their high energy density and reaction rate [1,2]. Due to the close interfacial contact between fuel and oxidizer at the nanoscale, the average distances of heat and mass transfer are drastically shortened, so that the reaction dynamics and energy release properties are significantly improved, such as faster energy release rates, shorter ignition delay times, lower ignition temperature [3,4,5], etc. Nanothermites are therefore often used in device integration and in the military sector, as well as as energetic additives in explosives and propellants [6,7,8,9]. They have sparked great interest in energetic materials.

Much of the research in nanothermites has focused on producing thermite systems with different shapes/geometries, i.e., multilayer [10], core–shell [11,12], and 3D porous nanostructures [13,14]. Among these forms, the core–shell structure has the largest contact area between fuel and oxidizer and the smallest diffusion length and time scale. Therefore, the core–shell nanothermite reactions can proceed as discrete solid particle combustion in the absence of gaseous oxygen, which has attracted great attention. For example, Huang et al. [15] prepared Si@Fe_2_O_3_ core–shell nanothermites by an electroless deposition method and found that the Si@Fe_2_O_3_ core–shell nanoparticles had a lower reaction onset temperature (~550 °C) than the mechanically mixed Si/Fe_2_O_3_ nanothermites (>650 °C). Feng and co-workers [16,17] used an atomic layer deposition (ALD) technique to prepare Al@Fe_2_O_3_ and Al@CuO_x_ core–shell nanocomposites and found that the core–shell Al@Fe_2_O_3_ has a lower onset temperature, a higher energy release and many times faster reaction speed than conventional Al/Fe_2_O_3_ nanopowders, and Al@CuO_x_ core–shell nanocomposites are reflected in a significantly shortened ignition delay time, oxidation temperature and significantly increased reaction speed. Wang et al. [18] fabricated energetic Si@CuO material with a core–shell structure by a self-assembly method. They found that the maximum actual heat release of Si@CuO with a core–shell structure is 1061.4 J/g, which is more than 532.8 J/g of energetic Si/CuO materials prepared by an ultrasonic mixing method. Shi et al. [12] adopted the alcohol thermal technique to synthesize Al@CuO nanothermite with an approximate core–shell structure. Compared with ultrasonic mixed Al/CuO, core–shell Al@CuO has a lower apparent activation energy of interfacial reaction in the solid, a higher light intensity, a shorter burning time, a larger pressure release, and a higher pressurization rate. Wen and colleagues [19] found that the core–shell particles produced in this way predominantly undergo a solid-state reaction mechanism, which has a 30% lower activation energy compared to physically mixed nanocomposites (215.0 vs. 310.8 kJ mol^−1^). All of these studies showed that the excellent reactive properties of nanothermites with a core–shell structure benefit from improved interfacial contact. However, these experimental studies mainly focus on the preparation methods and exothermic properties of nanothermites with metal core-metal oxide-shell structures, and studies on the reaction mechanisms of nanothermites with core–shell structure are still missing. In addition, few researchers have prepared the metal oxide core with metal shell nanothermites.

Our previous work [20,21] investigated the reaction mechanism and the influence of the atomic ratio of N_Al_/N_O_ on the thermal decomposition of Al core and SiO_2_ shell nanoparticles through molecular dynamics (MD) simulations. In this work, we study the reaction process of core–shell SiO_2_@Al nanoparticles under adiabatic conditions through MD simulations using a reactive force field (ReaxFF). The response properties were investigated by examining the time evolution of various physical quantities, including the number of Si-O, Al-O and Si-Si bonds, mean square displacement (MSD), radial distribution functions (RDF), snapshots and steric charge distributions, intermediate and final products.

## 2. Results and Discussion

### 2.1. Nanostructured Evolution

**Figure 1:**
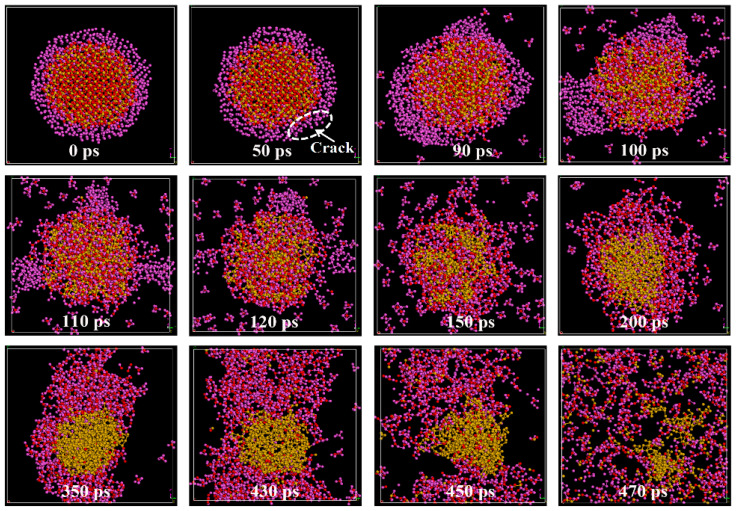
Snapshots of the *yz* cross-sectional view during the reaction processes of SiO_2_@Al, where the violet, red and yellow color particles denote Al, O and Si atoms, respectively.

To gain a comprehensive insight into the reaction process of SiO_2_@Al core–shell nanoparticles, the nanostructured evolution is investigated. Figure 1 shows the snapshots of the *yz* cross-sectional view at different reaction times during the reaction process of SiO_2_@Al under adiabatic conditions. The snapshot at 0 ps is the post-relaxation configuration as the initial structure of the reaction process. From the 50 ps snapshot in Figure 1, we can see that the energy released during the formation of the Al-O bond causes the Al shell to rupture upon melting. And the energy released during the redox reaction leads to a disorder of the SiO_2_ core, which starts at the interface and quickly moves inward as the reaction progresses, leading to the volume expansion of the nanoparticles. At 90 ps, small Al_n_O fragments are observed being ejected from the surface of the nanoparticles. Due to surface tension, the molten Al shell tends to aggregate into liquid droplets, as shown in the 100 ps snapshot. From the snapshots from 100 ps to 120 ps, it can be seen that liquid Al droplets accelerate the redox reaction with SiO_2_, the number of Al_n_O gas clusters increases, and Si is gradually reduced. We then observe the pure Si liquid droplets in the core at 200 ps, indicating that the redox reaction is completed. It is noteworthy that the number of Al_n_O gas clusters decreases from 150 ps. At 200 ps, the Al_n_O gas clusters adhere to the nanoparticles to form agglomerates. At the 350 ps snapshot, the nanoparticle structure remains in the form of pure Si droplets, liquid SiO compounds, and a small amount of Al_n_O gas clusters, indicating that the response of the system has reached dynamic equilibrium. At 430 ps, the Si droplets begin to decompose and the number of gas fragments increases, indicating that the nanoparticle is exploding. As seen in the 470 ps snapshot, the gas clusters fill the entire space, clearly showing that the system has exploded.

### 2.2. Gaseous Products

**Figure 2:**
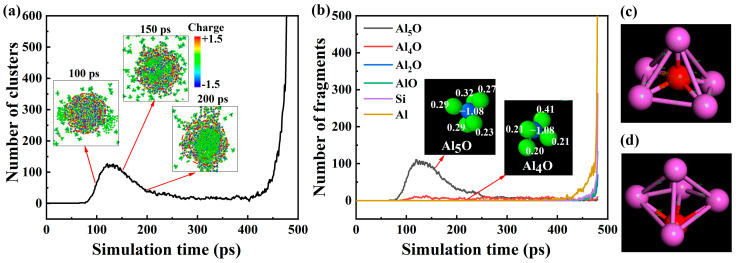
Time evolution of the number of (**a**) clusters and (**b**) fragments during the decomposition of SiO_2_@Al. The Al_5_O structure (**c**) obtained with ReaxFF, (**d**) optimized by the DFT method.

To further analyze the variation in the number of gas clusters observed in the snapshots of Figure 1, the number of gas clusters, intermediate products Al_5_O and Al_4_O, and final products Al_2_O, AlO, Si, and Al are counted at different times and shown in Figure 2. The species of the products is similar to the experimental results (Al_2_O, AlO, Al) [22] and another theoretical study (metal vapor and Al suboxides) [23] of Al/CuO. As can be seen in Figure 2a, the number of gas clusters increased rapidly after 80 ps, reaching the maximum at around 120 ps, then began to slowly decrease and remained stable. This is consistent with the phenomenon observed in Figure 1. Further analysis of the intermediates in Figure 2b revealed that the change in the number of gas clusters is mainly due to the amount of intermediate Al_5_O, and the amount of intermediate Al_4_O is almost constant at this time. This may be due to the interaction of electric field forces between the intermediates and the shell of the nanoparticles. The steric charge distributions at 100 ps, 150 ps, and 200 ps are also shown in Figure 2a. We focus on the charge distribution between two red-dotted circles. According to quantitative calculations, all atoms between two red-dotted circles carry 2.35|e| positive electricity at 100 ps. From the charge distribution of the Al_5_O and Al_4_O cluster in Figure 2b, it can be seen that the charge of the Al_5_O clusters is about 0.32|e|. As shown in Figure 1 and Figure 2b,c, we found that the structure of the Al_5_O clusters are mainly distorted pentahedron units. We further optimized their structures using the density functional theory (DFT) under the 0 K and ambient pressures and found that the final structures were mostly slightly deformed, as shown in Figure 2d, but these changes, whether in terms of geometric structure or total energy of the cluster itself, are all relatively small, indicating that the Al_5_O can indeed exist in the form of clusters. Of course, in extreme cases of high temperatures, the anharmonic vibrations between atoms in the cluster will lead to changes in the bond lengths between atoms, and these changes are inconsistent. That is to say, Al-Al bonds with metallic bonding properties will become longer, while Al-O bonds with covalent bonding properties will have relatively shorter changes, and therefore, the distortion of the cluster structure is completely understandable, as shown from Figure 2c,d. Due to the limitation of ReaxFF in treating the energetic material system at high temperatures [24], we can use AIMD to determine the existence and evolution of these clusters at different extreme temperatures, which can be discussed in detail in future work. But the tetrahedron Al_4_O cluster is nearly charge-neutral, at −0.05|e|, and is consistent with the results by Campbell and co-workers [25]. So, the Al_5_O clusters and the shell of the nanoparticles repel each other, and more and more Al_5_O clusters are emitted from the surface of the nanoparticles. At 150 ps, the total charge of the atoms between the two red-dashed circles is −4.84|e|. In the electric field generated by the shell of the nanoparticles, the positively charged Al_5_O clusters are attracted and then converge to the nanoparticles. This is the reason for the decreasing number of Al_5_O clusters after 120 ps observed in Figure 1 and Figure 2 after 120 ps. But at 200 ps, the charge between two red-dotted circles is very small, at 0.11|e|. From Figure 2a, it can be seen that the number of clusters stabilizes between 200 ps and 430 ps. After 430 ps, gaseous products rapidly form in large quantities. Figure 2b shows that the products are mainly metal vapor (Al and Si) and metallic metal suboxides (Al_2_O and AlO), which is consistent with the previous study [21,23].

### 2.3. Bond Analysis

**Figure 3:**
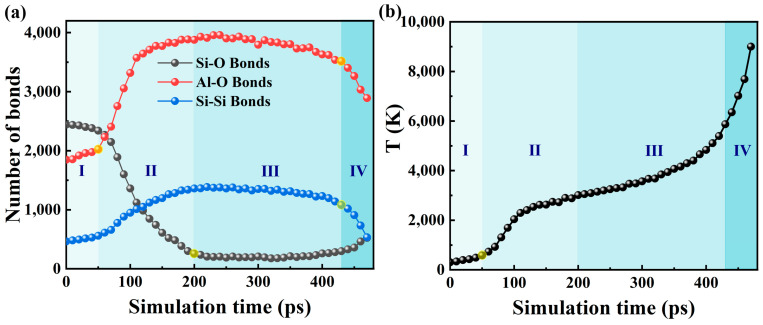
Temporal evolution of (**a**) number of Si-O, Al-O, and Si-Si bonds, and (**b**) temperature during the reaction processes of SiO_2_@Al.

To explain the reactions in more detail, we show the evolution of the number of different bond types and the temperature in Figure 3. Three turning points can be seen in Figure 3a, which divides the reaction process into four phases. The first stage shows a slight increase in the number of Al-O bonds and Si-Si bonds, and a slight decrease in the number of Si-O bonds. This indicates bond rupture of Si-O, and the bond formation of Al-O and Si-Si is relatively slow at this stage. Therefore, the heat released by the thermite reaction at the interface is very small, corresponding to a small temperature increase, as shown in Figure 3b. As the number of Al-O bonds increases, the first inflexion is encountered (yellow circle, at about 50 ps), corresponding to the ignition point. This can also be defined as the inflection point temperature in Figure 3b. After this point, many Al-O bonds are formed, so the temperature (580 K, see Figure 3b) at this point is considered as the ignition temperature, and time (50 ps) is considered as the ignition delay time of nanothermite. The second stage of the reaction shows a sharp decrease in the number of Si-O bonds until the second inflection point (yellow circle, at about 200 ps) indicates the rapid dissociation of the Si-O bonds and the completion of the exothermic redox reaction. At this stage, the bond formation of Al-O and Si-Si occurs relatively quickly at first and then relatively slowly, confirming the fast redox reaction observed before 120 ps in Figure 1. Figure 3b shows that the temperature increases rapidly before 120 ps and then increases slowly with time, further confirming the rapid exothermic reaction before 120 ps. However, the slow temperature increase after 120 ps is due to the amount of Al-O formed, and the heat released decreases. In the third stage, the number of Al-O, Si-Si and bonds is almost constant, indicating that bond breaking and bond formation have reached equilibrium. The snapshots in Figure 1 also intuitively confirm this point. Therefore, the temperature increases slowly during the simulation time, as shown in Figure 3b. The third inflection point (yellow circle, at about 430 ps) of the Al-O and Si-Si bonds indicates the detonation of the nanoparticles. Therefore, in the final phase, the bond rupture of Al-O and Si-Si progresses quickly, while the bond formation of Si-O progresses relatively slowly. The temperature increases rapidly over time.

### 2.4. Diffusion Analysis

**Figure 4:**
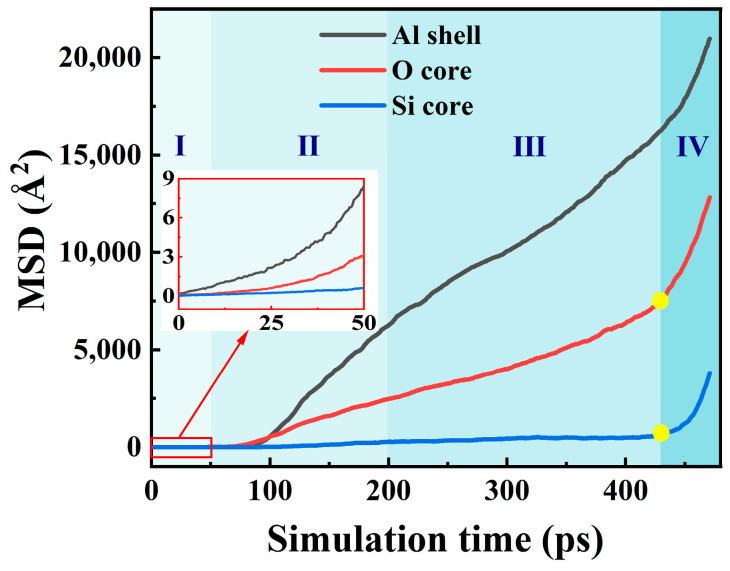
Temporal evolution of MSD during the reaction processes of SiO_2_@Al.

Our previous studies [26] concluded that the diffusion mechanism is the reaction mechanism of nanothermite, while the diffusion property is studied during the reaction processes of SiO_2_@Al. The basic parameter that characterizes diffusion is the diffusion coefficient D. MSD can provide the diffusion coefficient D, so the MSD is calculated for different atoms and shown in Figure 4. Using the Einstein relation, $D=\frac{1}{6N}\underset{t\to \infty }{\lim}\frac{d}{dt}\sum_{i=1}^{N}\left({\left|r_{i}(t)-r_{i}(0)\right|}^{2}\right)$, and $MSD=\frac{1}{N}\sum_{i=1}^{N}\left({\left|r_{i}(t)-r_{i}(0)\right|}^{2}\right)$, where *r_i_*(*t*) denotes the place of atom *i* at a given time *t*, and N is the number of atoms. The diffusion coefficients of Al, O, and Si atoms at the four stages can be calculated, and the results are shown in Table 1.

From Table 1, it can be seen that in the first stage (0–50 ps), all atoms show solid-state diffusion behavior. However, in the second stage (50–200 ps), the diffusion coefficient of Si is 3.24 × 10^−9^ m^2^/s, indicating that it has liquid diffusion behavior, while the diffusion coefficients of Al and O are 8.00 × 10^−8^ m^2^/s and 3.14 × 10^−8^ m^2^/s, respectively, indicating that Al and O atoms exhibit partly liquid diffusion behavior and partly gas diffusion behavior. This quantitatively illustrates that at this stage, the intermediate gas products were released from the nanoparticle surfaces observed in Figure 1 and Figure 2. It is noteworthy that Al in the third stage had a lower diffusion coefficient (200–430 ps) than in the second stage because most of the Al_5_O clusters adhered to agglomerates on the nanoparticles observed in Figure 1. In the last step (430–470 ps), the diffusion coefficients of the Al, O, and Si atoms are all in the 10^−7^ orders of magnitude, so that a transition from the liquid to the gas phase takes place at this stage.

### 2.5. RDF Analysis

**Figure 5:**
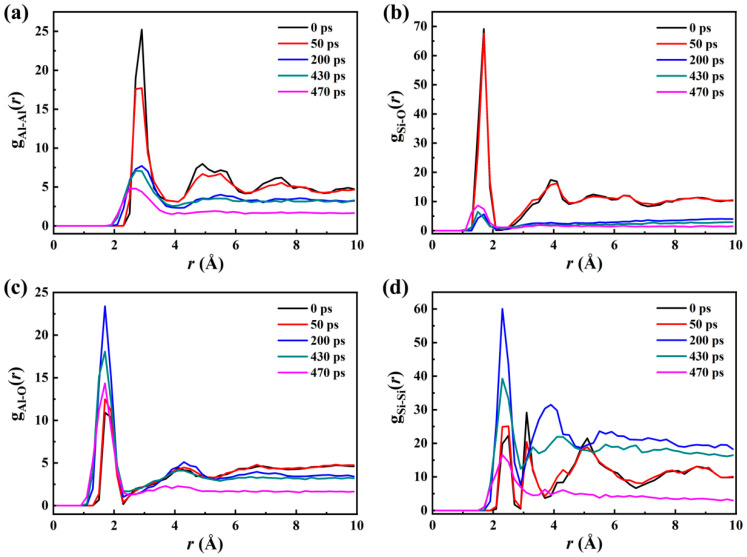
Comparison of partial radial distribution functions *g*(*r*) of (**a**) Al–Al, (**b**) Si–O, (**c**) Al–O and (**d**) Si–Si pairs at different times during the reaction processes of SiO_2_@Al.

To study the phase state of the system at different times, the partial radial distribution functions *g*(*r*) of Al–Al, Si–O, Al–O and Si–Si pairs are calculated and shown in Figure 5. The *g*(*r*) gives the local atomic arrangement and is a tool for distinguishing between solid, liquid and gas. The Al–Al atomic pairs in the SiO_2_@Al core–shell nanoparticles are shown in Figure 5a. At *t* = 0 ps, the first peak of *g*_Al–Al_(*r*) is sharp and strong, but *g*_Al–Al_(*r*) has few peaks and virtually no long-range order, indicating that the Al shell is an amorphous solid. At *t* = 50 ps, the Al–Al radial distribution function exhibits the shape typically found for a liquid diffusion peak (with fewer nearest neighbors, and the first peak becomes flat and weak) and no long-range order, indicating the shell has melted and is in the liquid state. After 200 ps, the intensity of the *g*_Al–Al_(*r*) peaks further decreases because of the decomposition of the Al shell, which formed the aluminum oxygen compounds observed in Figure 1. Notably, at *t* = 470 ps, except the broader and weaker first peak, all of the latter peaks disappear and become similar horizontal straight lines, indicating that most of the Al in the system is already in the gaseous state. According to Figure 5b, the first peak of *g*_Si–O_(*r*) occurred at *r* = 1.7 Å at 0 ps and 50 ps, and its sharp shape indicated strong interactions between Si and O atoms. As the reaction of aluminum and cristobalite progresses, the intensity of the first peak *g*_Si–O_(*r*) weakens (*t* = 200, 430 ps), and other peaks disappear, showing that the Si–O bonds are completely broken with no interaction, which is similar to the nanostructured evolution shown in Figure 1 and temporal evolution of number of Si–O bonds in Figure 3a. As can be seen in Figure 5c, the first peak intensity of *g*_Al–O_(*r*) increased significantly to the maximum at 200 ps as the Al atoms reacted with the O atoms of SiO_2_ in the course of the reaction, which is consistent with the conclusion obtained in Figure 1 and Figure 3a that the system undergoes a redox reaction that generates aluminum oxide. After 200 ps, the first peak intensity of *g*_Al–O_(*r*) began to decline, indicating that the interactions between Al and O atoms become weak. At *t* = 470 ps, the first peak of *g*_Al–O_(*r*) still retains its sharp shape, and the second peak of *g*_Al–O_(*r*) disappears, further illustrating that most of the final gas products found in Figure 2b are mainly aluminum metallic suboxides (Al_2_O and AlO). From Figure 5d, *g*_Si–Si_(*r*) has multiple peaks and practically long-range order at *t* = 0 ps and 50 ps, indicating that SiO_2_ core is a crystal. At *t* = 200 ps, the first peak of *g*_Si–Si_(*r*) is sharp and strong, but *g*_Si–Si_(*r*) has few peaks, confirming the pure liquid Si observed in Figure 1. At *t* = 470 ps, the curve of *g*_Si–Si_(*r*) is similar to the curve of *g*_Al–O_(*r*), which is another indication that most metallic Si products are already in the gaseous state.

## 3. Computational Details

All MD simulations were implemented in a large-scale atomic/molecular massively parallel simulator (LAMMPS) [27]. OVITO software (version 3.8.5) [28] was used to visualize the simulation snapshots and charge distribution. Interatomic interactions were described by the reactive force field (ReaxFF), which was proposed by van Duin, Goddard and collaborators in 2001 [29]. The ReaxFF parameters for Al/Si/O reported by Narayanan et al. [30] are composed of original Si/O parameters [31], Al/O parameters [32] and trained Al/Si parameters. This parameter set has been successfully used to describe the reactivity between Al/Si/O atoms, and its feasibility and accuracy for the Al/SiO_2_ systems have been validated in previous works [20,21,29,33]. The principle is to describe the interaction of chemical systems and the formation of bonds by determining the bond energy level according to the distance between atoms.

**Figure 6:**
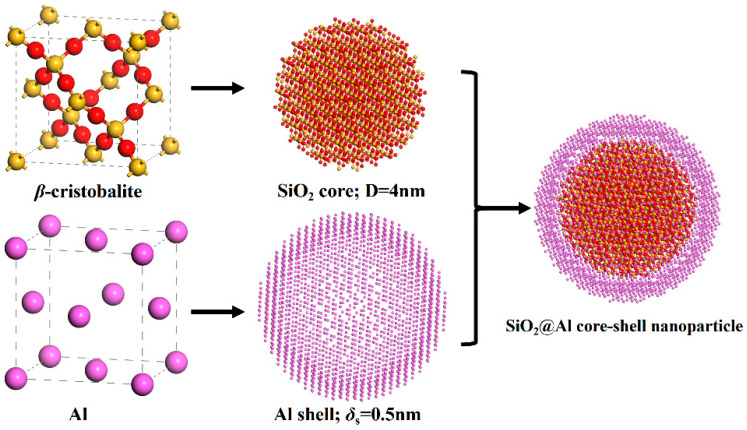
Construction of core–shell structured SiO_2_@Al nanoparticle. Coloring denotes the type of atom: yellow, Si atom; red, O atom; violet, Al atom.

The original configuration of the core–shell SiO_2_@Al nanoparticle consisted of a nanocrystalline β-cristobalite (SiO_2_) core with a diameter of 4 nm containing 2193 atoms and a nanosized face-centered cubic (fcc) crystalline aluminum shell with 0.5 nm 2204 atoms. The spatial distances between the SiO_2_ core and the Al shell were chosen to be approximately 0.2 nm, so that the total diameter of the core–shell SiO_2_@Al nanoparticle is 5.4 nm, with a total of 4397 atoms (2204 Al + 729 Si + 1464 O). The SiO_2_@Al nanoparticle was placed in the center of a cubic box with sides of 6.88 nm to model the reaction process in a relatively free space. The details of the models are shown in Figure 6. It is important to note that there is always a naturally generated aluminum oxide layer of approximately 2 nm on the Al at room temperature [34]. However, the purpose of this work is to increase the high reactivity of the pure Al surface and improve the reaction rate of SiO_2_@Al. To avoid the interference of Al oxides, the active Al content in the SiO_2_@Al models used in this study is up to 100%.

**Figure 7:**
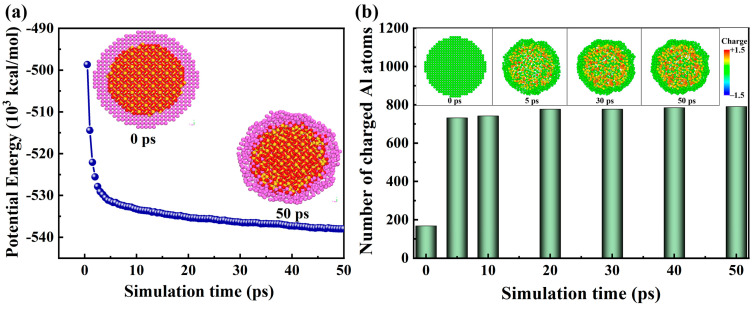
(**a**) Temporal variation of SiO_2_@Al potential energy, and (**b**) number of charged Al atoms and the cross-sectional view of the charge distribution of the Al shell in the SiO_2_@Al at different times during relaxation in the NVT ensemble.

In this study, all MD simulations used the periodic boundary condition and a time step of 1 fs. The initial configuration of the SiO_2_@Al nanoparticle was first minimized in energy using the conjugate gradient (CG) algorithm. The second stage was subjected to a relaxation phase to eliminate the residual stress in the nanoparticle so that the energy of the nanoparticle reaches its lowest state. The SiO_2_@Al nanoparticle was relaxed at 300 K for 50 ps in the NVT ensemble. The temporal variation of potential energy during relaxation is shown in Figure 7a. The relative error of potential energy at *t* = 30~50 ps is 0.29%, which is below the criterion of structural stability (the fluctuation of potential energy within 20 ps is less than 1.2%) [35]. After 50 ps, the system reached a stable state. The *yz* cross-sectional view of the relaxed configuration is also shown in Figure 7a. Compared to the pre-relaxation configuration, the relaxed Al shell has more irregular atom arrangements and the coupling between the Al shell and the SiO_2_ core is tighter, resulting in a more stable structure. Therefore, the duration of the relaxation phase corresponds to the requirements. Figure 7b shows the number of charged Al atoms and charge distribution of the Al shell in the SiO_2_@Al nanoparticle at different times during the initial relaxation phase. It can be observed that the oxide film has already formed at the inner interface of the Al shell after 10 ps, which is due to the reactivity between pure Al and SiO_2_, leading to redox reactions and charge transfer. This also explains the significant decrease in potential energy. The third stage was carried out in the microcanonical (NVE) ensemble to simulate the adiabatic thermite reaction up to thermal decomposition. The reaction simulations lasted 470 ps.

## 4. Summary

This study investigates the reaction processes of core–shell SiO_2_@Al nanoparticles in a microcanonical ensemble using MD and ReaxFF. The results show that the Al shell cracks and melts into droplets in the first stage. Then, Al and SiO_2_ undergo intense redox reactions to form pure Si. At the same time, intermediate products Al_5_O and Al_4_O are found in this stage. Due to the coulomb force interaction between the intermediate products and the shell of the nanoparticle, a majority of Al_5_O clusters are first ejected from the system and then adhere to agglomerates on the nanoparticles. In the next stage, the diffusion coefficient of Al becomes smaller, and bond breakage and bond formation have reached a dynamic equilibrium. At last, the decomposition of Si droplets and the formation of final products Al_2_O, AlO, Si and Al occur, suggesting the full decomposition of the nanoparticle. This work can complement the complex reaction mechanism of nanothermite.

## Figures and Tables

**Table 1:** Diffusion coefficients of Al, O, and Si atoms at four reaction stages.

Stages	0–50 ps	50–200 ps	200–430 ps	430–470 ps
D_Al_ (m^2^/s)	2.41 × 10^−10^	8.00 × 10^−8^	6.88 × 10^−8^	1.89 × 10^−7^
D_O_ (m^2^/s)	9.28 × 10^−11^	3.14 × 10^−8^	3.42 × 10^−8^	2.12 × 10^−7^
D_Si_ (m^2^/s)	1.78 × 10^−11^	3.24 × 10^−9^	1.95 × 10^−9^	1.16 × 10^−7^

## Data Availability

The original contributions presented in the study are included in the article, further inquiries can be directed to the corresponding authors.
